# Longitudinal bond strength of a universal adhesive and chemical dentin characterization under different acid etching protocols

**DOI:** 10.1590/1678-7757-2023-0359

**Published:** 2024-02-26

**Authors:** Lucélia Lemes GONÇALVES, Anuradha PRAKKI, Tânia Mara da SILVA, Arwa BAFAIL, Janaína BORTOLATTO, Alexander Terry STAVROULLAKIS, Sérgio Eduardo de Paiva GONÇALVES

**Affiliations:** 1 Universidade Estadual Paulista Instituto de Ciência e Tecnologia Departamento de Odontologia Restauradora São José dos Campos Brasil Universidade Estadual Paulista (UNESP), Instituto de Ciência e Tecnologia da UNESP, Departamento de Odontologia Restauradora, São José dos Campos, Brasil.; 2 University of Toronto Faculty of Dentistry Dental Research Institute Toronto ON Canada University of Toronto, Faculty of Dentistry, Dental Research Institute, Toronto, ON, Canada.; 3 Taibah University College of Dentistry Department of Restorative Dental Sciences Madinah Saudi Arabia Taibah University, College of Dentistry, Department of Restorative Dental Sciences, Madinah, Saudi Arabia.

**Keywords:** Dentin, Acid etching, Adhesives, Matrix metalloproteinases, Collagen

## Abstract

**Objective:**

This study aimed to analyze the longitudinal bond strength of a universal adhesive and chemically characterize the dentin substrate under different acid etching protocols.

**Methodology:**

Dentin samples were etched with polyacrylic acid 25% (PAA) for 10 seconds (n=3) and phosphoric acid 32% (PA) for 15 seconds (n=3) and analyzed by Fourier transform infrared spectroscopy – attenuated total reflectance (FTIR-ATR) before and after treatment. For collagen degradation, samples (n=12) were divided into 3 groups: PAA, PA, and Deionized water (control), and analyzed by the quantity of solubilized type I collagen C-terminal cross-linked telopeptides and solubilized C-terminal peptide in relation to total protein concentration (ICTPtp and CTXtp) and by their ultimate tensile strength (UTS). For the adhesive interface analysis, dentin samples (n=72) were divided into 3 groups: PAA, PA, and Self-etch (SE), and subdivided into 2 groups: 24 h (baseline) and 1 year. The following tests were performed: microtensile bond strength (μTBS) (n=48), scanning electron microscopy (SEM) (n=12), and nanoleakage (n=12).

**Results:**

The FTIR of PAA showed lower reduction of the peaks in the phosphate group when compared to PA. For ICTPtp, PA showed a significantly higher value. For CTXtp, PA and PAA groups failed to statically differ from each other. UTS was significantly lower for PA. For μTBS, storage time significantly affected bond strength. The results were unaffected by the etching protocol. For SEM, after 1 year, PA had little evidence of degradation in the upper third of the adhesive interface in comparison to the other groups. Nanoleakage showed no considerable silver impregnation after 1 year in the SE group.

**Conclusion:**

The use of PAA prior to a universal adhesive (when compared to PA) represents a less aggressive type of etching to dentin. However, self-etching still seems to be the best option for universal adhesive systems that have functional monomers in their composition.

## Introduction

The bonding between an adhesive and dentin is considered a form of tissue engineering based on a reaction of material exchange described by the so-called “Adhesion-Decalcification concept.”^1^ Within this concept, the dentinal mineral phase is removed by acids and a porous collagen scaffold is exposed. Minerals are replaced by resin monomers that envelope the collagen fibrils forming after polymerization, the principal mechanism for dentinal adhesion, well-known as the hybrid layer, which is mainly responsible for stable long-term dentin bonding.^2-5^

The hybrid layer is obtained by partially or totally removing minerals with acidic primers in self-etch adhesives or acid etching in etch-and-rinse adhesive systems.^1,5^ Recently, more versatile adhesives have been introduced, called universal adhesives, which can be adapted to both strategies.^6^ The latter adhesive systems usually contain functional monomers which are capable of chemically interacting with hydroxyapatite, contributing to bond durability.^1,4^ However, in the long term, the adhesive interface is vulnerable to hydrolytic degradation,^6^ to mechanical stress, and to the activity of endogenous enzymes such as matrix metalloproteinases and cysteine cathepsins.^3^ In this sense, the role of endogenous enzymes has stood out among other factors.^7^Nevertheless, the demineralization process by acids can potentially promote enzymatic activation and initiate the process of collagenolytic degradation.^8,9^

Self-etch adhesives containing acidic monomers simultaneously demineralize and infiltrate the dental substrate, with no requirement of an initial acid etching step with phosphoric acid, preserving the dentinal calcium phosphate.^1^ Etch-and-rinse procedures promote the complete removal of the smear layer, demineralization, and calcium depletion, which may adversely affect the potential chemical bond between the dentin and adhesive system.^6,10,11^ As an alternative protocol, polyacrylic acid, which is commonly used as a mild dentin conditioner to improve glass ionomer cement (GIC) adhesion and is associated to self-etch cements, can be applied instead of phosphoric acid.^8,12^ Polyacrylic acid is less aggressive but able to etch dentin, partially removing the smear layer. This generates sites for hybridization, leaving considerable amounts of hydroxyapatite on the collagen surface and increasing the potential for chemical interaction with reduced potential to activate matrix metalloproteinases (MMPs).^6^

The aims of this study were to: A) chemically characterize the dentin substrate by FTIR before and after etching protocols; B) measure dentin collagen degradation under different etching protocols by matrix degradation by MMPs and cathepsin-K (CAT-K), respectively. These were measured by quantification of solubilized type I collagen C-terminal cross-linked telopeptides (ICTP), solubilized C-terminal peptide (CTX), and the ultimate tensile strength (UTS); and C) to analyze the longitudinal bond strength interface of a universal adhesive subjected to different acid etching protocols (*i.e*., self-etch, phosphoric acid, and polyacrylic acid) after 24 hours (baseline) and 1 year of water storage. These were measured by microtensile bond strength test (μTBS), scanning electron microscopy (SEM), and nanoleakage by SEM. The tested null hypotheses were (1) different etching protocols that would neither affect dentin protease activity nor (2) different etching protocols and storage times that would affect the quality of the adhesive interface.

## Methodology

### Specimen preparation

**Figure 1:**
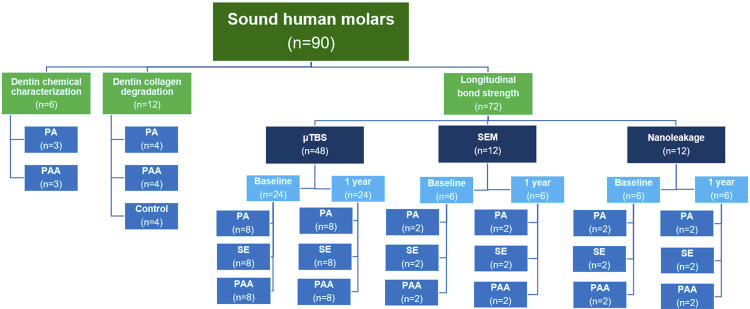
Methodology workflow

This study was approved by the local Institutional Review Board (Protocol 1.784.538). In total, 90 sound human molars were collected and stored in distilled water at 4º C after the removal of soft tissues for a maximum period of 6 months until use. The study methodology is illustrated by a workflow presented in Figure 1.

### Chemical characterization of the dentin substrate (FTIR)

For this test, 6 specimens obtained from sound human molars were analyzed. The teeth were fixed into an acrylic resin holder and sectioned using a low-speed laboratory cutting machine (Labcut 1010, Extec Technologies Inc., CT, USA) under water cooling. Overall, two horizontal sections were performed: parallel to the occlusal surface to expose the mid-coronal dentin and 1 mm below the enamel-cementum junction to separate the crown from the roots, which were discarded. The pulpal tissues were removed with curettes. The dentin surfaces were polished using 600-grit aluminum oxide abrasive discs (Extec Corp., CT, USA) in a polishing device (DP-10, Panambra, SP, Brazil) under water cooling to standardize the smear layer. Next, half of the specimens were etched with 32 % phosphoric acid for 15 s (PA) and the other half with 25% polyacrylic acid (PAA) for 10 s. Chemical analysis was evaluated by FTIR-ATR (PerkinElmer Waltham, Massachusetts, USA). All specimens were analyzed before and after the etching procedure. The spectra were acquired in absorbance mode under a frequency range from 4000 to 650 cm^-1^, with 32 scans at a resolution of 4 cm^-1^. After normalization, the absorbance peaks of phosphate (from 900 to 1200 cm^-1^), carbonate (870 - 1070 cm^-1^), amide groups from the dentin collagen matrix (between 1600-1700 cm^-1^), and proline and hydroxyproline (1454 cm^-1^)^13^ were compared between substrates.^14^ The integrity of the collagen triple helix was performed by analysis of the ratio of the absorbance of the 1235 (amide III) and 1450 cm^-1^ (stereochemistry of the pyrrolidine rings) bands. Therefore, if this value is close to 0.5, the integrity of the collagen triple helix is compromised. If the ratio is around 1, the integrity of amide III and C-H bond of the pyrrolidine ring of the type I collagen triple helix is maintained.^15^

### Dentin collagen degradation analysis: Specimen preparation

For the dentin collagen degradation tests, the enamel and superficial dentin of 12 sound human molars were removed by horizontal sectioning 1 mm below the central fissure using a slow-speed diamond saw under water cooling (Isomet, Buehler Ltd, Lake Bluff, IL, USA). The teeth were sectioned at the middle third of their crowns perpendicular to their long axis, with a thickness of 0.5±0.1 mm, by means of a slow-speed diamond saw at 300 rpm under water cooling. Dentin disks were completely demineralized in 10% phosphoric acid (H_3_PO_4_) (LabChem, Zelienople, PA, USA) at room temperature under agitation for 40 h using a hematology chemistry mixer (Model 346; Fisher Scientific Co., Toronto, ON, Canada) at a speed of 12 rpm.^16^ Dentin matrices were then thoroughly rinsed in deionized water for 10 min prior to subsequent analyses. After demineralization, the matrices were dried in a desiccator for 24 h, and the baseline dry mass was recorded using an analytical balance (Denver PI-214A Analytical Semi-micro Balance, Denver Instrument Company, Denver, CO, USA). The disks were equally distributed into 3 experimental groups (*n*=4): 1) PA, 2) PAA and 3) Deionized water treatment (control). After the dry mass measurement, the disks were completely rehydrated in deionized water for 1 hour to recover their original dimensions and treated with the respective etching procedures (Table 1). The control group consisted of demineralized dentin disks that were kept in deionized water for 30 s. The matrices were immersed in 50 mL of buffered medium (pH 7.2) for 5 min to neutralize the acid etchants and then blot-dried. Each disk was then immersed in 1 mL of a calcium-and-zinc-containing incubation medium in labeled polypropylene tubes. The medium contained 5 mM HEPES, 2.5 mM CaCl_2_-H_2_O, and 0.05 mM ZnCl_2_ (pH 7.2).^16^

**Table 1:** Materials, composition, and application procedure

Materials	Composition	Procedure
Scotchbond Universal Etchant 3M ESPE, St Paul, USA.(665021)	32% phosphoric acid by weight	Apply 15 s., water rinse 15 s.
Ketac™ Conditioner 3M ESPE, St Paul, USA. (656054)	25% polyacrylic acid	Apply 10 s., water rinse 15 s.
Scotch Bond Universal 3M ESPE, Sumaré, Saint Paul, Brazil. (507329)	15-25% BisGMA, 15-25% HEMA, 5-15% dimethacrylate resins, 5-15% silano, 1-10% monomer phosphate MDP, 1-5% Vitrebond™ copolymer (polyalckenoic acid), 10-15% ethanol, 10-15% water, <2% primers, in mass.	Apply for 20 s, according to the manufacturer’s instructions. Gently air dry for 5 s. Light cured for 10 s (LED Light Curing System, Demi Plus, Kerr Corporation, WI, USA)
Filtek Z350 XT, 3M ESPE, Sumaré, Saint Paul, Brazil.(828241)	BisGMA, BisEMA UDMA, TEGDMA, PEGDMA, nanoparticles of zircônia/silica.	Light cured for 20 s (LED Light Curing System, Demi Plus, Kerr Corporation, WI, USA)

*Abbreviations: Bis-GMA: bisphenol A diglycidyl methacrylate; HEMA: hydroxyethyl metacrylate; MDP: 10-methacryloxydecyl dihydrogen phosphate; Bis-EMA: bisphenol A diglycidyl methacrylate ethoxylated; TEGDMA: triethylene glycol dimethacrylate, UDMA: urethane dimethacrylate; PEGDMA: polyethylene glycol dimethacrylate.

### Total protein and solubilized telopeptide assays

Aliquots of 1 µL randomly selected from the different media were used for total protein evaluations (*n*=4). Total protein concentrations were measured using the Protein A280 Nanodrop™ 1/Onec Microvolume UV-Vis Spectrophometer (Thermo Scientific, Wilmington, DE, USA) at 280 nm. Matrix degradation by MMPs was measured by the quantity of solubilized type I collagen C-terminal cross-linked telopeptides (ICTP), using 2 aliquots of 100 µL from different media and groups (*n*=4) and the ICTP ELISA kit (MyBiosource Inc, San Diego, USA). To determine matrix degradation by cysteine cathepsins, the amount of solubilized C-terminal peptide (CTX) was quantified using 3 aliquots of 100 µL from different media and groups (*n*=4) and the CTX ELISA kit (MyBiosource Inc, San Diego, USA). The average ratios of ICTP and CTX in relation to total protein concentration (ICTPtp and CTXtp) were calculated.^16^ Mean values for ICTPtp and CTXtp were analyzed by 1-way ANOVA followed by Tukey’s *post-hoc* test. For all tests, the level of significance was set at 0.05.

### Ultimate tensile strength (UTS) of demineralized dentin

For UTS, 36 demineralized dentin beams were obtained from 12 previously tested demineralized etched samples. The specimens measured approximately 1 mm width × 0.5 mm thickness. Beams were glued to a jig using cyanoacrylate adhesive and an accelerator (Zapit, Dental Ventures of America, CA, USA) with microbrush applicators, which was mounted on a horizontal microtensile testing machine (Micro Tensile Tester, Bisco Inc., TX, USA) and subjected to tension force at a crosshead speed of 0.5 mm/min using a 10 kgf load cell until failure. Strength values were recorded (N), after which mean values were calculated for each group (MPa). The UTS values from the beams of the same tooth sample were averaged and the mean strength was used as 1 unit for statistical analysis.^16^ Mean values for UTS were analyzed by 1-way ANOVA followed by Tukey’s *post-hoc* test. The level of significance was set at 0.05.

### Adhesive interface analysis by μTBS, SEM, and nanoleakage by SEM: specimen preparation

In total, 72 teeth were fixed in an acrylic cylindrical holder (2.5 cm/diameter and 2 cm/height) with dental wax and sectioned by a cutting machine under water cooling (Labcut, Extec; Enfield, CT, USA). Teeth were sectioned at the middle third of their crowns perpendicular to the long axis, with a thickness of 0.5±0.1 mm by means of a slow-speed diamond saw (Isomet, Buehler Ltd., Lake Bluff, IL, USA) at 300 rpm under water cooling. To produce and standardize the smear layer, the dentin surfaces were polished using 600-grit aluminum oxide abrasive disks (Extec Corp., CT, USA) in a polishing device (DP-10, Panambra, São Paulo, Brazil) under water cooling. The specimens were randomly divided into 3 groups according to the acid etching protocol (*n*=24): 1) PA, 2) PAA, and 3) Self-Etch for 20 s (SE). Each group was randomly divided into 2 subgroups (*n*=12) based on the period of storage: 24 h and 1 year. The Scotch Bond Universal (SBU) adhesive system was applied according to the manufacturer’s recommendations. Resin composite blocks (4 mm/diameter and 2 mm/height) were built up on the pre-treated dentin. The specimens of the baseline group were immersed in distilled water at 37 °C for 24 h prior to testing (Table 1). The specimens of the one-year group were stored for 12 months in distilled water with periodic changes of the storage solution once a week. Antibiotics were not used.

### Microtensile bond strength and fractographic analysis

Overall, 48 restored specimens were used for the microtensile bond strength test (μTBS). For the baseline test, the specimens were sectioned into dentin-resin composite beams (0.8 mm^2^) that were suitable for μTBS using a cutting machine (Labcut, Extec; Enfield, CT, USA) under water cooling 24 hours before the test in accordance with the storage time. The beams were individually stored for 24 h in identified tubes (Eppendorf, São Paulo, Brazil) containing deionized water at 37 °C. Before the μTBS test, the dimensions of the beam sections were measured at the adhesive interface area with a digital caliper (Starret Industria e Comercio; Itu, SP, Brazil). Each beam was fixed with cyanoacrylate glue and an accelerator (Zapit, Dental Ventures of America, CA, USA) with microbrush applicators to a metal jig for the mechanical test using a universal testing machine (EMIC DL-1000, Equipamentos e Sistemas Ltda., Paraná, Brazil) at a crosshead speed of 0.5 mm/min and a 10 kgf load cell. The mean value (in MPa) for the beams originating from each tooth (n=8) was calculated and used for statistical analyses. The failure modes were analyzed under a stereomicroscope (Karl Zeiss, Oberkochen, Baden-Württemberg, Germany) using 50× magnification and classified as cohesive failure involving about 75% dentin or resin composite, adhesive failure of about 75% resin/dentin interface, or mixed failure at the resin/dentin interface. Only the adhesive and mixed failures were included in statistical analyses.^17^ Data showed the normal distribution of the means and were analyzed using two-way ANOVA (independent variables: etching protocol and storage time) and the Tukey’s test. Level of significance was set at 5%.

### Qualitative analysis of the adhesive interface by Scanning electron microscopy (SEM)

For the qualitative analysis, two specimens of each group were prepared for hybrid layer evaluation under SEM (*n=*12). After the restorative procedures, the teeth were sectioned perpendicularly to the adhesive interface. The sections were polished using 2400- and 4000-grit aluminum oxide abrasive disks (Extec Corp., CT, USA) in a polishing device (DP-10, Panambra, São Paulo, Brazil) under water cooling. All specimens were etched with 32% phosphoric acid without silica (32% Uni-Etch, Bisco) for 15 s and rinsed for 10 s. Next, the specimens were dehydrated, sputter-coated with gold/palladium, and the adhesive interfaces were examined in a scanning electron microscope (Inspect S50, FEI, Hillsboro, Oregon, EUA) at 15kV using 2000× magnification.^18^

### Nanoleakage by SEM

For the nanoleakage analysis, two specimens of each experimental group were prepared (*n=*12). After restorative procedures and storage times, the samples were immersed in ammoniacal silver nitrate which was prepared according to the previously described protocol.^19^ The samples were placed in ammoniacal silver nitrate in total darkness for 18 h, rinsed thoroughly in distilled water, and immersed in a photo-developing solution for 6 h under a fluorescent light to reduce silver ions into metallic silver grains within the voids along the adhesive interface. The penetration of silver nitrate into the adhesive interface and hybrid layer were evaluated by the analysis of 2000×-magnification SEM micrographs and graded in accordance with the percentage of the affected area by nanoleakage: no leakage = score 0, slight leakage = score 1 (<25%), moderate leakage = score 2 (25% ≤ 50%), and distinct leakage = score 3 (>50%).^20^ The specimens were treated as described above for the SEM analysis.

## Results

### Chemical characterization of the dentin substrate (FTIR)

**Figure 2:**
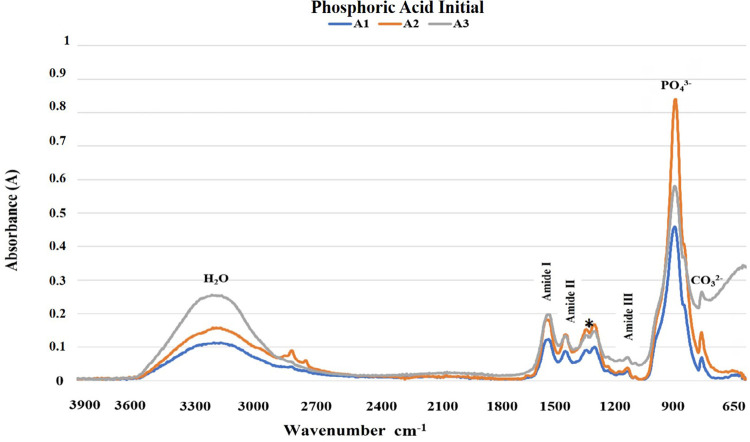
Absorption spectra in the infrared region from 3900 to 650 cm-1 (A1, A2, and A3) of the human dentin prior to the phosphoric acid etching protocol (H2O: water; PO43-: phosphate group; CO32-: carbonate group; *: pyrrolidine rings (proline and hydroxyproline) region).

**Figure 3:**
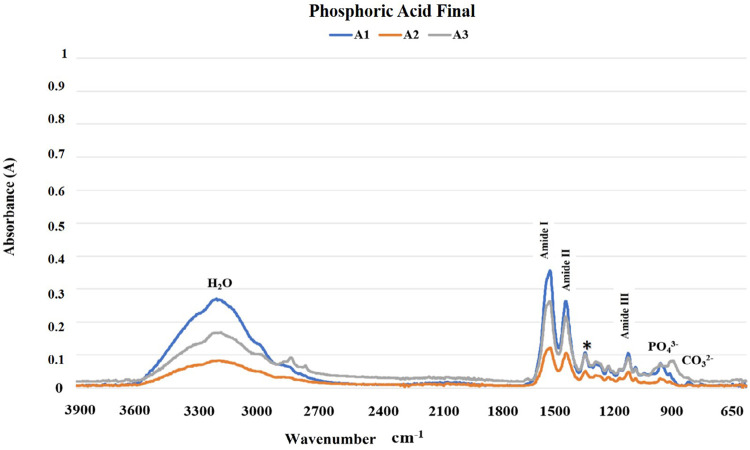
Absorption spectra in the infrared region from 3900 to 650 cm-1 (A1, A2, and A3) of the human dentin after the phosphoric acid etching protocol (H2O: water; PO43-: phosphate group; CO32-: carbonate group; *: pyrrolidine rings (proline and hydroxyproline) region).

**Figure 4:**
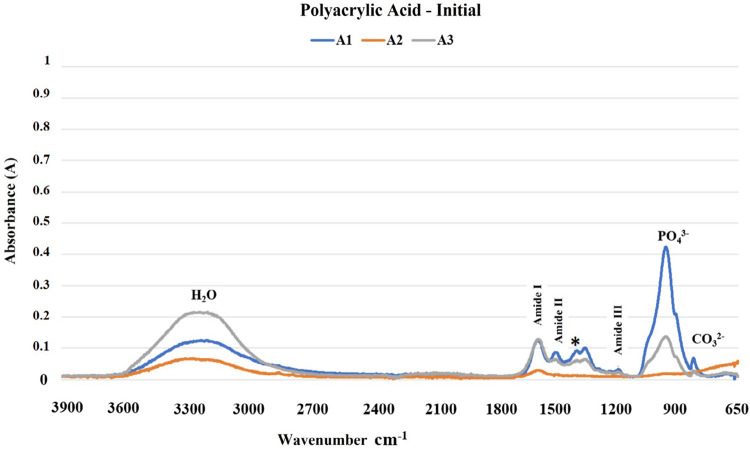
Absorption spectra in the infrared region from 3900 to 650 cm-1 (A1, A2, and A3) of the human dentin prior to the polyacrylic acid etching protocol (H2O: water; PO43-: phosphate group; CO32-: carbonate group; *: pyrrolidine rings (proline and hydroxyproline) region).

**Figure 5:**
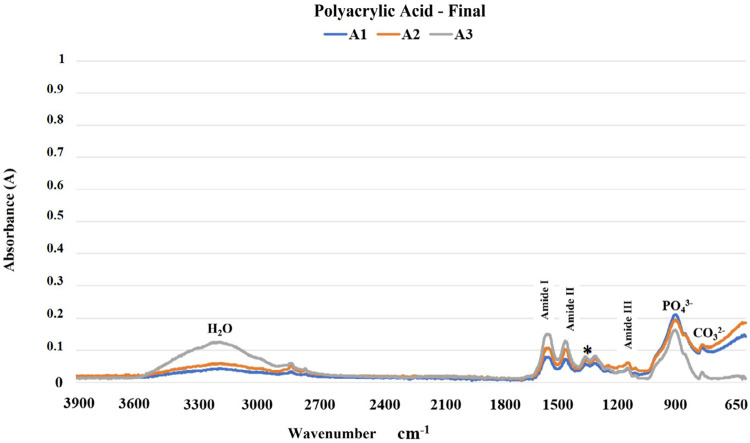
Absorption spectra in the infrared region from 3900 to 650 cm-1 (A1, A2, and A3) of the human dentin after the polyacrylic acid etching protocol (H2O: water; PO43: phosphate group; CO32-: carbonate group; *: pyrrolidine rings (proline and hydroxyproline) region).

The results of the FTIR analysis are shown in Figures 2-5. The mineral content of the dentin etched with PA decreased when compared with the sound dentin (Figure 2) as identified by the reduction of peaks in the phosphate and carbonate groups. Moreover, there was an increase in the peaks of the amide groups present in the collagen matrix with a slight change in the absorption intensity of the amide III bands and pyrrolidine rings (Figure 3). The use of PAA showed lower reduction of the peaks in the phosphate group when compared to PA. The carbonate, amide, and pyrrolidine ring groups seemed to have an increase in absorbance peaks (Figures 4-5). For all experimental conditions, the analysis of integrity of collagen triple helix showed no collagen denaturation since the means of the absorbance ratios around 1235 cm^-1^ and 1450 cm^-1^ (pyrrolidine ring) were close to the unit PA (0.95) and PAA (0.80).

### Dentin matrix degradation analysis: Total protein and solubilized telopeptide assays and UTS of demineralized dentin

ICTPtp and CTXtp mean protein concentrations and UTS values are presented in Table 2. The statistical analysis for ICTPtp showed that there were statistical differences between PA, PAA, and control group mean values (*p*<0.05). The PA group had the significantly highest value of ICTPtp, which statistically differed from the PAA, and the control group had the lowest value. Moreover, the statistical analysis for CTXtp showed that the PA and PAA group failed to statically differ from each other. However, both differed from the control group (*p*>0.05). Tensile strength value was significantly lower for the PA when compared to the PAA and control groups. The PAA failed to significantly differ from the control group (*p*>0.05).

**Table 2:** Mean values for the total protein concentrations of ICTPtp, CTXtp, and UTS

Groups	ICTPtp	CTXtp	UTS
	**(s.d.)**	**(s.d.)**	**(s.d.)**
PA	62.17^A^	1.13^A^	1.03^A^
	(8.64)	(0.32)	(0.20)
PAA	45.68^B^	0.74^A^	1.65^B^
	(4.46)	(0.18)	(0.33)
Control	1.55^C^	0.02^B^	1.84^B^
	(0.38)	(0.005)	(0.15)

*Standard deviation: α=0.05. Same letters within columns indicate no statistical difference.

### Microtensile bond strength and fractographic analysis

**Figure 6:**
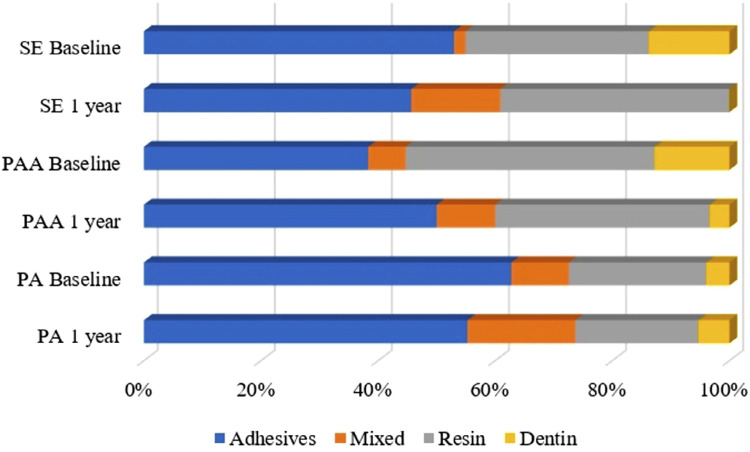
Failure mode percentages obtained in all groups after the microtensile test. SE: Self-etch; PAA: Polyacrylic acid; PA: Phosphoric Acid

Although the factor storage time significantly affected bond strength (F=38.49; *p*=0.0001), results (Table 3) were unaffected by the etching protocol (F=0.74; *p*=0.48) and showed no interaction between factors (F=1.29; *p*=0.28). They also showed no significant differences between storage times regarding different etching protocols. According to the fractographic analysis (Figure 6), the adhesive/mixed fractures were the most abundant at baseline in the PA and SE groups, whereas PAA showed a higher occurrence of cohesive failures in resin and dentin. After one year of storage, PA and PAA showed an increase in the incidence of adhesive/mixed failures, whereas SE showed a higher percentage of resin cohesive failures.

**Table 3:** Mean bond strength ± standard deviation values

Groups	Baseline	1 year
PA	40.13 ± 8.94 MPa^Aa^	30.06 ± 8.32 MPa^Ab^
PAA	43.51 ± 4.95 MPa^Aa^	24.35 ± 7.08 MPa^Ab^
SE	45.52 ± 8.91MPa^Aa^	30.35 ± 6.66 MPa^Ab^

*Standard deviation: α=0.05. Same upper-case letters within columns and lower-case letters within rows indicate no statistical difference.

### Qualitative analysis of the adhesive interface by SEM

**Figure 7:**
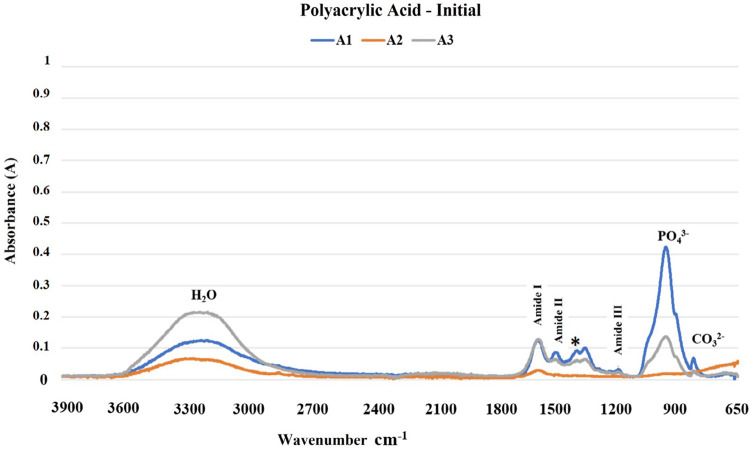
SEM micrographs of the adhesive–dentin interface etched with PA. Both baseline (A) and 1-year (B) images showed signs of degradation and numerous resin tags, whereas the PAA images showed different patterns between both periods. At baseline, deep penetration of adhesive was discernible by resin tags. However, after one year, an increase of degradation in the upper third of the adhesive interface was noticeable (C, D). The SE group showed few and small resin tags with remarkable signs of degradation (E, F). (A: adhesive layer; R: resin composite; D: dentin; arrow: resin tags; pointing finger: signs of degradation).

The SEM results in Figure 7 show vertical sections of the adhesive interface. The comparative analysis of the adhesive interface for the PA group in both periods showed that the adhesive interface after one year of water storage still had numerous resin tags and that the adhesive layer integrity showed little evidence of degradation in the upper third of the adhesive interface, i.e., the area that is in contact with the resin restoration (A, B). The PAA group showed changes across different storage periods. After one year, there was a decrease in the number of resin tags as well as signs of degradation in the upper third of the adhesive interface (C, D). The SE group adhesive interface at baseline showed few and small resin tags, a characteristic profile of self-etching adhesive systems. The analysis of the interface after one year of storage showed a reduction in the adhesive layer with signs of degradation in the upper third of the adhesive interface (E, F).

### Nanoleakage by SEM

**Figure 8:**
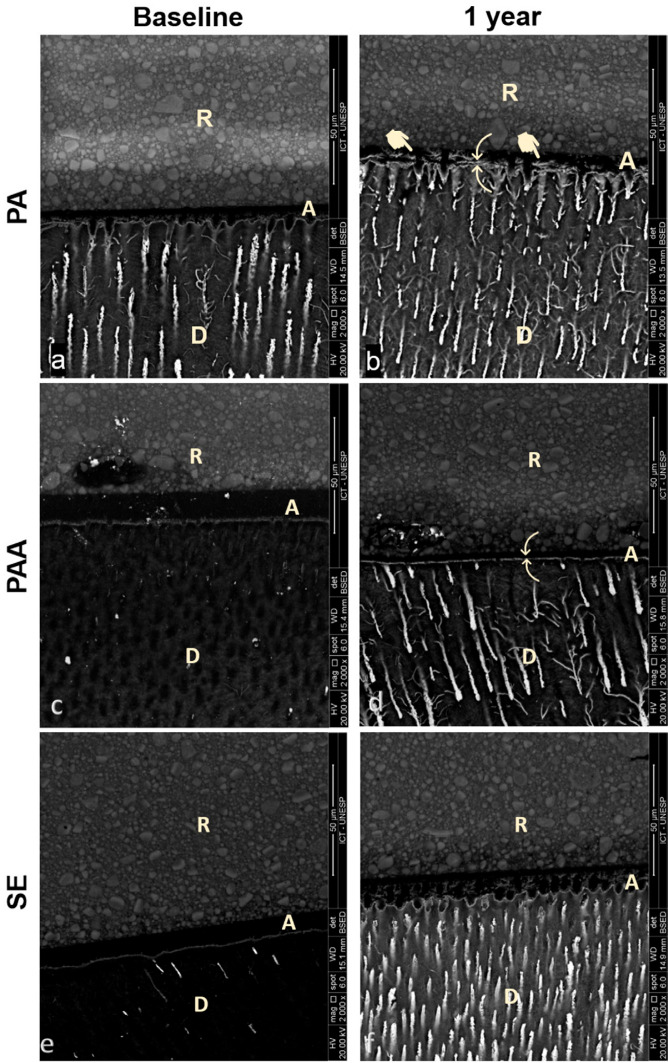
SEM micrographs of the adhesive-dentin interface of nanoleakage analysis. The adhesive interface of the PA group showed a considerable rate of nanoleakage after 1-year storage, and more than 50% of the interface showed a sign of nanoleakage (score 3). No indication of nanoleakage was detected (score 0) at baseline (a, b). The same pattern was detected in the PAA group (c, d). However, for the SE group, none of the storage periods showed significant signs of nanoleakage, and the index remained the same without changes in score (0) (e, f). (A: adhesive layer; R: composite resin; D: dentin; arrow: length of the silver nitrate uptake; pointing finger: accumulation sites of silver nitrate).

Representative SEM images of the resin–dentin interfaces for all etching protocols are depicted in Figure 8. None of the treatments at baseline exhibited significant signals of silver nitrate. The PA group at baseline (a) showed no evidence of nanoleakage (score 0). However, after one year of storage (b), more than 50% of the interface showed a sign of nanoleakage (score 3). For the PAA group at baseline (c), no evidence of nanoleakage was detected (score 0). However, after one year (d), the interface exhibited more than 50% nanoleakage in the evaluated area (score 3). The SE group at baseline (e) showed no nanoleakage (score 0). And after one year of storage(f), the index remained the same without changing the score (0). There was no considerable silver impregnation after one year in the SE group.

## Discussion

The study of the physicochemical characteristics of dentin is essential for understanding adhesion mechanisms since the formation of resin-dentin interface involves a series of treatments on the substrate that completely change its properties.

The results obtained by the chemical characterization of the substrate help clarify the controversies associated with collagen denaturation after acid etching protocols.^21^ For both acids, following the etching protocols, the analysis of the ratio between the absorbances of the Amide III bands (1235 cm^-1^) and pyrrolidine rings (1450 cm^-1^) were about one unit, which indicates no structure denaturation.^13,15^ Although both acid etching protocols failed to affect the collagen helical structure, the differences between the chemical interactions of the substrate and the acids are evident (Figures 2-5). Phosphoric acid performs a more aggressive etching to the dentin, exposing a large amount of collagen fibers due to drastic mineral removal. On the other hand, PAA acts as a mild etching agent, exposing collagen fibers more superficially and maintaining a greater amount of phosphate and carbonate in the structure.^6^ This characteristic promotes chemical adhesion to the substrate while protecting the collagen fibers. During the bonding procedure, dentin etching is performed to expose collagen fibers and facilitate the formation of the hybrid layer. However, the incomplete encapsulation of collagen fibers by resin monomers can generate degradation sites at the adhesive interface.^22,23^ Additionally, exposure of the collagen extracellular matrix to endogenous or exogenous stimuli, such as the use of acids, can cause changes in the regulatory mechanisms of endogenous proteases.^22,24^

In this study, quantification of dentin collagen degradation by the application of different acid etching protocols was performed by indirectly measuring the activity of MMPs and Cathepsin K (CAT-K). According to our results (Table 2), H_0_1 was rejected since the acid etching treatment affected the activity of MMPs and CAT-K. The results showed that the PA group presented higher release of ICTP fragments, which corroborates studies that indicate the activity of MMPs and reduced tensile strength of dentin collagen, factors that can contribute to longitudinal degradation.^9,25,26^ Conversely, the results of this study suggest that PAA strategy seems to be less aggressive regarding the activation of MMPs and UTS of demineralized dentin.^8^ The literature presents controversial results regarding the ability of phosphoric acid to promote the activation of MMPs, suggesting that, in some cases, it could promote the reduction of collagenolytic activity by MMPs and CAT-K denaturation.^24^

There were no differences between PA CTX_tp_ and PAA CTX_tp_ concentrations, which suggests that both acids have the same potential to activate CAT-K. The control group analysis showed a low concentration of ICTP_tp_ and CTX_tp_, which indicates dentin protease activity even in the absence of an etching protocol. This could likely have been caused by the 10% phosphoric acid initially used to demineralize dentin. Moreover, Pashley, et al.^24^ (2004) have also demonstrated that collagen can undergo degradation over time even under aseptic conditions due to the action of the intrinsic proteases present in the matrix. This effect has been related to mechanisms other than acidic treatment that can also induce protease activation.^8^ Furthermore, activation of certain latent MMPs can be induced by active MMP-2 or Cysteine-cathepsins.^27^

According to the UTS results of demineralized dentin, the use of PA significantly affected the tensile strength of the dentin matrix when compared to the PAA and control groups. Therefore, it is possible that the use of PA may affect not only the activity of endoproteases but also the integrity of collagen itself.^25^ However, the findings of this study strongly suggest that the use of phosphoric acid has greater potential in activating MMPs when compared to PAA. This study seems to be the first to associate the use of a universal adhesive to the PAA etching protocol, which makes it difficult to establish direct comparisons. However, it is well known that the main factors associated with adhesive interface degradation are the collagen matrix longitudinal biodegradation and the hydrolysis of the hybrid layer.^28^

The microtensile strength test showed no statistical difference regarding the analyzed etching strategies. The comparison between all groups suggests that the experimental etching with PAA was comparable to the conventional ones (PA and SE) from a mechanical point of view. The partial smear layer removal and dentin demineralization caused by the PAA and SE may have positively affected the performance of the adhesive system. This led functional monomers to interact with the hydroxyapatite by ionic interactions with the carboxyl groups of the polyalkenoic acid, contributing to chemical adhesion^6^ and further longevity. Although chemical bonding fails to translate into higher bond strengths, it may prevent bond strength reduction upon aging.^1^

Our results led to the partial rejection of the null hypothesis H_0_2 since the storage period factor significantly affected results (*p*>0.0001) (Table 3). All groups showed a significant reduction in microtensile bond strength over time. Therefore, regarding the interaction between the dentin complex substrate and the etching modes and based on our results, we agree with researchers who continue to acknowledge the self-etch approach.^6,29^

Fractographic analysis (Figure 6) showed higher adhesive and mixed failure modes for the PA and SE groups in both storage periods.^30^ However, the PAA group showed a high incidence of resin and dentin cohesive failures, respectively, for the immediate analysis time. Nevertheless, after one year, the PAA fracture pattern changed to adhesive and mixed failures. These results may suggest that unlike PA and SE, PAA may improve the adhesive bond strength over time, which can be explained by an improved long-term chemical adhesion. SEM results showed that all etching protocols had signs of longitudinal degradation (Figure 7), which agreed with the bond strength results. It is established that signs of adhesive interface degradation with reduction of the adhesive layer and the quantity and size of resinous tags can serve as sites of continuous longitudinal degradation. Moreover, defects present in the interface may serve as sites of nanoleakage.^31^

In this study, nanoleakage rates were compared using scores. This methodology has been extensively used to demonstrate the quality of adhesive interface and their chronic degradation patterns.^32^ The analysis of the PA, PAA, and SE for the immediate time (Figures 8 a,c,e) showed a score of 0, indicating the absence of nanoleakage. However, after one year of storage, PA and PAA (Figures. 8 b, d) showed nanoleakage in more than 50% of the evaluated area, whereas SE (Figure 8 f) showed no significant signs of nanoleakage for this period. The same result was found by Marchesi, et al.^30^ (2014), which showed lower longitudinal nanoleakage for SE when compared to PA. This performance may be attributed to the presence of functional monomers in the composition of the adhesive as the protective action of 10-MDP exerted on the fibers of collagen.^33^ MDP-Ca salt is formed by the reaction between MDP and hydroxyapatite. It may mechanically protect demineralized dentin collagen, preventing the invasion of exogenous proteases and MMPs and decreasing collagenase activity.^4,23^ Additionally, SBU is composed of Vitrebond Copolymer (VCP), a type of methacrylate-modified polyalkenoic acid copolymer, which also chemically binds to dentin.^6^ This result indicates that the additional etching step for dentin is more aggressive in terms of demineralization when compared to the SE strategy, consequently affecting its sealing capacity over time. However, in certain clinical contexts, particularly when astringent solutions are used for moisture control, the use of acid etching remains the most appropriate approach.^34^

## Conclusion

The use of 25% polyacrylic acid prior to the application of SBU can be indicated since it presents similar results to the conventional adhesive protocols. However, the fact that the this protocol showed no superior performance makes it a less attractive choice when compared to conventional ones. Notwithstanding, based on the results of this and previous studies, we agree that dentin self-etch approach still seems to be the best option for universal adhesive systems. More studies are necessary in order to investigate the influence of the polyacrylic acid on mineralized dentin after the application of universal adhesives, the activation of MMPs and cysteine cathepsins using different compositions of universal adhesive systems, and performing analyses for longer storage times.
